# Some Preliminary Notes on an Empirical Test of Freud’s Theory on Depression

**DOI:** 10.3389/fpsyg.2013.00158

**Published:** 2013-05-07

**Authors:** Mattias Desmet

**Affiliations:** ^1^Department of Psychoanalysis and Clinical Consulting, Faculty of Psychology and Educational Sciences, Ghent UniversityGhent, Belgium

**Keywords:** mourning and melancholia, depression, narcissistic identification, conceptual analysis

## Abstract

A review of the literature indicates that empirical researchers have difficulty translating Freud’s theory on depression into appropriate research questions and hypotheses. In their attempt to do so, the level of complexity in Freud’s work is often lost. As a result, what is empirically tested is no more than a caricature of the original theory. To help researchers avoid such problems, this study presents a conceptual analysis of Freud’s theory of depression as it is presented in Mourning and Melancholia (Freud, 1917). In analyzing Freud’s theory on the etiology of depression, it is essential to differentiate between (1) an identification with the satisfying and frustrating aspects of the love object, (2) the inter- and an intrapersonal loss of the love object, and (3) conscious and unconscious dynamics. A schematic representation of the mechanism of depression is put forward and a research design by which this schema can be empirically investigated is outlined.

## Introduction

A review of the empirical research into Freud’s theory on depression reveals two major problems. First, there is often a dramatic gap between Freud’s theory and the hypotheses empirical researchers develop from it. Most illustrative of this is the way in which Freud’s theoretical ideas about the association between depression and experiences of loss have been put to the test. In this context, Fisher and Greenberg (1996, p.26) observe that “with minor exceptions, researchers have chosen to define loss in terms of loss of significant family figures (primarily parents) usually due to death.” However, even a superficial reading of Freud’s (1917) ‘Mourning and Melancholia’ would suffice to show that his notion of loss is situated at a different level. Freud states that “In melancholia, the occasions which give rise to the illness extend for the most part beyond the clear case of a loss by death, and include all those situations of being slighted, neglected, or disappointed, which can import opposed feelings of love and hate into the relationship or reinforce an already existing ambivalence” (Freud, 1917, p. 251). For Freud there is no reason to believe that a loss – be it by death, divorce, or any other reason – should necessarily lead to depression if it is not connected to a feeling of being slighted or disappointed, and if it does not introduce a profound ambivalence toward what is lost. Fisher and Greenberg’s (1996, p. 29) review of the empirical research into the association between depression and loss concludes that “The symptoms of a major segment of those who become clinically depressed have not, in any empirical fashion, been shown to have been triggered by a loss as such.” What is ironic about Fisher and Greenberg’s (1996) observations, however, is that they are put forward as part of an argument *against* the validity of Freudian theory.

The second problem with empirical research into Freud’s theory of depression is that it virtually always focuses on isolated associations between depression and theoretically related variables, such as loss, narcissistic identification, feeling slighted, introjection of aggression, ambivalence, etc. (e.g., Fisher and Greenberg, 1996, pp. 24–25). It is clear, however, that in an isolated state, these variables are potentially related to a variety of other types of psychopathology as well. For example, ambivalence can be associated with obsessional neurosis, feeling slighted with hysteria, narcissistic identification with psychosis. As we will show in this study, Freud’s (1917) text clearly indicates that isolated expressions of these different characteristics should not be thought of as typical of depression; what Freud emphasizes is the occurrence of these different characteristics *in a specific constellation*.

Overall, the complexity of Freud’s theory is such that empirical researchers appear to have had difficulty translating it into appropriate hypotheses and research questions. Without a thorough understanding of Freud’s work, however, any hypotheses and research questions borne out of it will be overly simplistic. This means that testing such hypotheses will not reflect a test of the theory he put forward. One of the biggest challenges for psychoanalysis is reducing the gap between theory and empirical research. Indeed, conceptual and empirical research are too often carried out in separate spheres. Nevertheless, all research should start from theory and hopefully return to it. Without a robust theoretical framework empirical research has no compass and it is not long before one loses track of the phenomena under investigation.

In this study I present a conceptual analysis of Freud’s (1917) Mourning and Melancholia that can serve as a starting point for qualitative and quantitative research. In the course of my analysis, I draw from my own clinical practice to elaborate certain aspects of Freud’s theory. This clinical component of my analysis mainly consists of unsystematic observations and is introduced only as an elaboration of certain of Freud’s ideas. The conditions in which these observations were made are not explicitly outlined in this paper. As discussed below, in future research, the theoretical structure put forward here should be contrasted with clinical data gathered and analyzed in a systematic way. A research design that would be appropriate in this regard is presented in the final part of this study.

## Conceptual Analysis of Freud’s Theory on Depression

Above we argued that Freud conceived the experience of object loss in depression in a broader sense than the mere loss of a loved one through death. Such an interpretation not only misunderstands how Freud conceptualized the *nature* of the experience of loss, but also erroneously implies that loss is the *cause* of depression. Such causal reasoning is nowhere to be found at this stage of Freud’s work, where the reader will find his theoretical ideas are far more complex.

The first thing to note about Freud’s line of reasoning in Mourning and Melancholia is that loss is first experienced at the *inter*personal level and is, in a second moment, repeated at the *intra*personal level. The first loss is situated in relation to a loved one. Here he states that “An object-choice, an attachment of the libido to a particular person, had at one time existed; then, owing to a real slight or disappointment coming from this loved person, the object-relationship was shattered” (Freud, 1917, pp. 248–249). This slight turns a quantum of love into hate and thus entails ambivalence toward the loved person. As a consequence of this conflict, the loved one is dropped. Freud does not go into further detail about the nature of this “slight or disappointment.” However, my own clinical observations suggest that the slight is often *passive* in nature. The love object failed to act when the patient was in a situation where she/he needed help. This situation is usually associated with a broader Oedipal constellation: in the narratives of patients affected by severe depression, there is usually a polarized description of the parents. One of them is described as a tyrant, who oppressed both the other parent and the patient. The other parent is usually described as a victim; weak and unable to defend him/herself. The narratives of such patients also often refer to the tyrant-parent as the one who is the cause of all suffering. In this context, it is not infrequent that the patient gives accounts of having been done some kind of great injustice by him/her. The other parent – who is usually in the position of love object – is often described as having been too weak to intervene. The therapeutic process often reveals that at an unconscious level, this failure was the trigger for the depression. Thus, with respect to the “slights and disappointments” outlined in Freud’s etiology of depression, we must distinguish between the conscious and the unconscious level. At the conscious level, we find a certain injustice inflicted by the tyrant-parent; at the unconscious level we find the passiveness of the victim-parent.

As stated above, Freud did not consider such slights and disappointments to be the *cause* of depression. The cause is rather situated at the level of a specific way of responding to such disappointments. After being (seriously) disappointed by the love object, most people transfer their libidinal investment to another person, i.e., they replace their love object with another. However, people vulnerable to depression react in another way. They withdraw the libido from the object to the ego and reinvest it in two ways. On the one hand, it is used to erect a *narcissistic identification* with the love object. On the other hand it is invested in sadistic impulses operating in the super-ego which attack the identification with the love object in the ego by means of harsh self-reproaches. Finally, the sadism prevails and the libidinal investments of the representations of the object in the ego are given up. This is the second loss, the intrapersonal loss, which is a loss of libido in the ego. Below I describe the different aspects of the process that leads to this intrapersonal loss in more detail, starting with Freud’s theory on the mechanism of identification.

In Freud’s view, identification always occurs in relation to a love object. People identify with something because it offers a certain advantage in the struggle for a love object. In Lacanian terms, we might say that people identify with something – a signifier – because it gives them a place in the desire of a loved other. Freud distinguishes between two types of identification. Hysterical identification – which is the most common – is an identification *with the possessor of the love object*. In other words, to have success in the competition for the desire of a loved other, one identifies with someone who already proved to be successful. This is the basic mechanism in hysterical epidemics. Let’s take the example of a school where all pupils are suddenly afflicted with stomach aches. When the first pupil falls ill, apparently due to the consumption of contaminated food in the school restaurant, the others – without having eaten the contaminated food – might unconsciously interpret the reactions of compassion extended to the affected pupil as a sign of love. Consequently, they begin to experience the same symptoms. The second type of identification is more unusual and concerns an identification *with the love object itself*. Freud (1921, p.108) gives the example of a boy who is closely attached to his mother and who, upon being sent to boarding school, starts taking care of the other children in the same way as his mother took care of him. Thus, to deal with the loss of the love object, he *becomes* the love object. According to Freud, this is what happens in depression. Upon being disappointed by a love object, the patient identifies with it. Freud does not specify, however, with what aspects of the love object the patient identifies. On the basis of my clinical experience, however, I put forward the hypothesis that it concerns an identification with the slighting, disappointing aspects of the object. I argued above that the disappointment in depression is passive in nature: the depressed person feels distress because the love object does not take the desired action. The depressive state – which is essentially a state of deep passivity and refusal to react to the stimuli of life – always boils down to an identification with the passiveness of the love object. The patient reacts to the failure of the love object according to the “an eye for an eye, a tooth for a tooth” principle (i.e., “if you don’t do anything for me, then I won’t do anything for you either”).

This sheds light on the subsequent step in Freud’s description of the etiology of depression. After identifying with the love object, sadism from the super-ego is directed at the newly formed identifications. However, this does not seem to be entirely correct: the sadism from the super-ego does not so much attack the *new* identification with the love object, but attacks the identification with the object that was made *before* the disappointment, when the relationship was predominantly satisfying. In that period, the patient installed narcissistic identifications with the tender, loving, and caring aspects of the love object. This is perhaps the reason why many depressive patients show excessively altruistic characteristics before the depression manifests, making them over-represented in care-giving occupations. In other words, the mental process of the depressed individual boils down to this: “Because you disappointed me by being passive in a situation in which I needed you, I will take revenge by becoming passive as well, and I won’t love or care for you any longer.”

Thus, what is lost at the intrapersonal level is the identification with the positive characteristics of the love object that was made prior to the disappointments. What is “gained” in this process is a set of identifications with the negative aspects of the love object. This has certain consequences. First, the depressive person feels worthless, a failure with respect to his/her altruistic ideals (stemming from the initial identification with the love object). Consequently, the depressive person starts to reproach himself/herself over a series of things that apply to the negative aspects of the love object. Here we are perfectly in line with Freud’s theory again, which explicitly states that self-reproaches are actually reproaches made toward the love object. Indeed, Freud remarks that such reproaches always simultaneously address identifications with the love object in the ego *and* the love object itself. This love object is often found to be in close proximity to the patient and is more or less burdened by the depression of the patient (Freud, 1917, p. 251). The destructive effect of depression on the people surrounding the affected individual has been documented extensively in empirical research (e.g., Coyne, 1976) and is one of the most characteristic aspects of Freud’s theory. In the end, Freud states, depression always boils down to sadism: by being unwell, the depressive patient attempts to torture the former love object, punishing him/her for his/her failures.

The exact mechanism of depressive sadism is not described in detail by Freud, but it is easy to see that it operates via the mechanism of *commiseration*. The depressive knows that if he/she suffers, the love object will suffer with him/her. That is why suffering becomes an attractor. Here we can refer to the critical analysis of Christianity that Nietzsche (1895) makes in *The Antichrist*. Christianity declared commiseration to be the fundamental virtue. Yet, according to Nietzsche, it is a vice rather than a virtue because, on the one hand, it multiplies suffering (when one person commiserates with a suffering person, two people suffer) and, on the other, because it makes suffering attractive (because it has an impact on the other). In the final analysis, therefore, the epidemic of depression in the Western industrialized world might be partially a consequence of centuries of cultivating commiseration as the highest virtue.

It’s important, however, to differentiate between an imaginary and a symbolic dimension in commiseration. The symbolic dimension is better indicated by the term *compassion*. Commiseration then implies that one sympathizes with the imago’s of suffering by which a depressive presents himself to the world. As such, it renders permanent those imago’s and it is therefore associated with resistance and stagnation in the depressive state (Lacan, 1953a,b). Compassion, however, implies a readiness to take a subject’s speech seriously, to confirm the inevitability of suffering within the parameters of the subjectivity of the patient, and to recognize the subjective strainedness in the discourse of the other. It’s this openness to speech that can be expected to transform the patient’s subjective structure and depressive experiences. As such, compassion is essential to the dénouement of the knot of depression.

Furthermore, a striking difference can be observed between conscious and unconscious mechanisms in depression. With respect to the slight, at the *conscious* level depressive complaints address the aggressive oppression of the tyrant-parent. At the *unconscious* level depressive complaints address the passiveness of the victim-parent. A similar distinction can be observed in the individual’s self-reproach. At the *conscious* level, aggression (from the super-ego) is directed toward the passive aspects of the ego. The content of the self-reproach always boils down to the fact that she/he is sick and passive, unable to do anything for loved ones. At the *unconscious* level, however, aggression is clearly directed toward the positive, caring aspects of the love object. Because the depressive feels slighted by the passiveness of the love object, she/he refuses to offer love and care any longer. In this light, the self-reproach that one *cannot* care anymore has to be qualified as false. Thus, depression reveals the fundamental falseness of the human ego. Or, as Lacan (1963, p. 79) puts it in the tenth seminar, it testifies to the fact that the human subject is essentially an entity that erases its own traces.

Before integrating the results of our analysis into a schema, we will discuss one other aspect of Freud’s (1917) Mourning and Melancholia. In this text, Freud does not specify whether the love object he is referring to is a love object from childhood or from the time when the patient becomes depressed. In line with the Freud broader theoretical frame, it seems clear that he is referring to both. In childhood, the future depressive’s representation of the love object was outlined above, i.e., at the conscious level the love object is represented as weak but loving, while at the unconscious level the love object is represented as disappointing and passive. Since these representations are invested with libido, the future depressive will love others who match these representations. This means that this set of representations – of signifiers in Lacanian terms – will pre-program a repetition of the same disappointment in later life. As soon as this becomes manifest, the whole mechanism described above is effectuated and inevitably leads to the depressive phenomenology. This should not imply that from time to time this deadly repetition cannot be halted, usually in an encounter with someone who is able to convince the depressive person that there is another way to exist for an Other.

**Figure 1:**
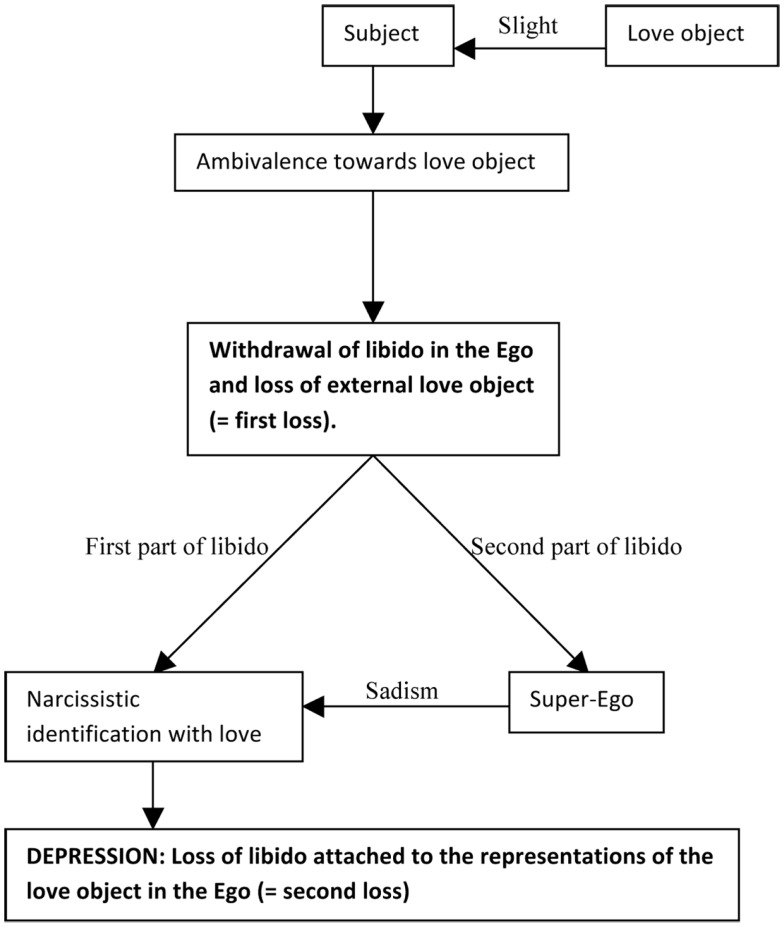
**The intra- and interpersonal loss in the etiology of depression according to the theory of Freud in Mourning and Melancholia**.

**Figure 2:**
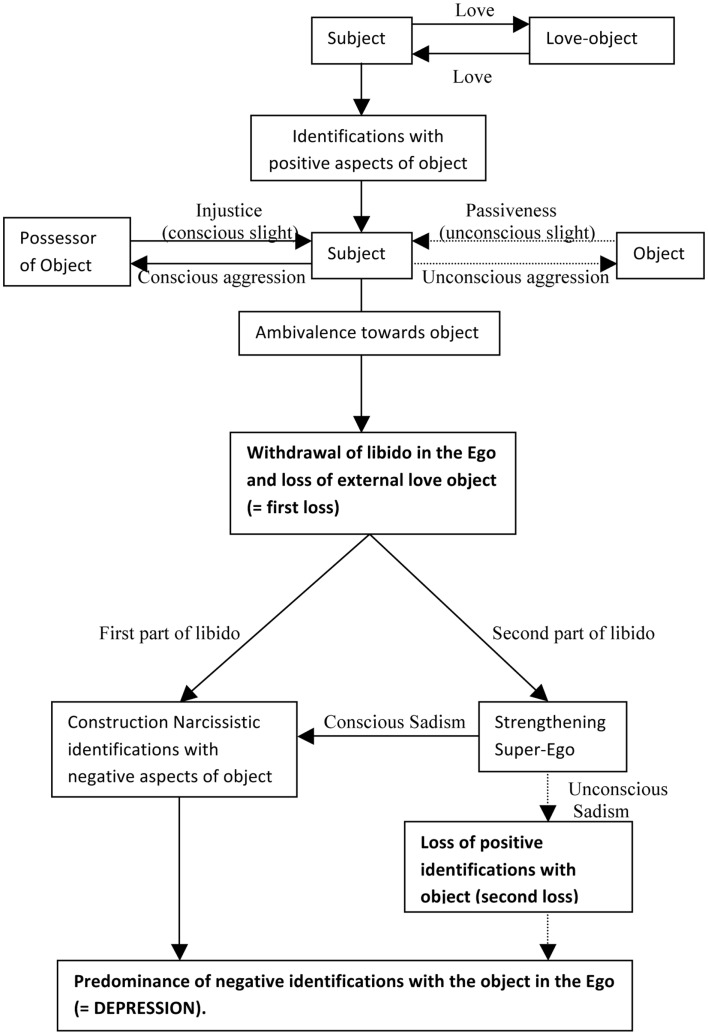
**The intra- and interpersonal loss in the etiology of depression differentiating between identifications with positive and negative aspects of the object**.

This was, in a nutshell, Freud’s theory on depression. Figure 1 presents the logical structure as it is presented by Freud in Mourning and Melancholia. Figure 2 presents the same logical structure extended with the elaborations presented above.

## Research Designs Appropriate to Test Freudian Theory on Depression

As was argued in the introduction, it is the totality of the mechanism presented in Figures 1 and 2 that should be tested in empirical research, not isolated components of it. None of the constituent parts can be deemed typical of depression. Hypotheses focusing on the different constituent parts (e.g., “Depressives systematically report feeling slighted by the passivity of their love objects”) can be formulated, but a global assessment of the structure described above is still needed.

The research design most appropriate for the assessment of this clinical complexity is without a doubt the naturalistic single case-study design. In naturalistic single case-study research the therapeutic process is studied in detail and in such a way that it interferes minimally with daily practice. This, of course, does not mean that the act of investigation leaves no traces: such research is suitable only for certain patients, where the therapist can reasonably believe that the research component will not interfere with the therapeutic process. In permissible cases, therapy sessions are recorded with the informed consent of the patient. Throughout the treatment, psychological and biological variables are measured, and medication use and health-care expenses are documented. Contrary to what is often believed, recording sessions proves to have little or no inhibiting effect on patients’ speech (Kächele et al., 2009). Psychometric evaluations are limited to self-report questionnaires, assessing “general distress,” and the patient’s specific symptoms. These assessments take no more than a few minutes and are conducted after every session. Few studies on the validity of psychometric measurements in single case-study designs have been carried out until now. However, it can be anticipated that for most variables included in single case-study designs, psychometric measurements will be far more precise compared to group designs. Important sources of measurement error, such as differential interpretations of scales between subjects and spontaneous fluctuations in the variables under investigation, are absent in single case-study research or can be controlled for.

In addition to the therapy recordings and the self-report data, once per month saliva samples are taken for four consecutive days, each morning and evening. The samples are gathered by the patient him/herself, at home, by chewing on a piece of wadding and placing it into a small tube (a so-called *salivette*). In these saliva samples, evolutions in the concentrations of hormones, such as cortisol, testosterone, and androstenone, can be registered through mass spectrometry. Receptor sensitivity on the genetic material in the saliva can also be determined. Gathering saliva samples is a non-intrusive and reliable method of investigating the biological effects of psychotherapy. When the therapy is complete, the therapy recordings are transcribed verbatim and the researcher can carry out an analysis of the data.

The ultimate goal of the analysis is evaluate the global structure of depression. In doing so, we start from the following assumptions: (1) That the theoretical structure specified above determines depressive complaints; (2) That the patient will progressively become aware of this structure during the therapeutic process and that this structure will appear more and more explicit in his/her speech; and (3) That depressive complaints will decrease, *not* at the moment the patient becomes aware of the depressogenic structure, but at the moment he/she distances him/herself from it, i.e., at the moment he/she replaces identifications with the passive/destructive aspects of the love object with identifications with active/constructive aspects of the love object. Merely gaining insight into this structure might well intensify the patient’s complaints; in most cases, a substantial period of working-through – in which the depressive identifications are progressively restructured and replaced by other identifications – will be needed.

In the first step of the analysis, fragments of the clinical material that refer to each of the separate elements of the theoretical structure (e.g., feeling slighted by a love object, ambivalence toward love object, etc.) are marked in the transcripts of the recorded sessions. As the therapeutic process progresses, we hypothesize that the patient will progressively integrate all of the separate elements into a comprehensive narrative surrounding his/her depression. Therefore, qualitative assessment is used to determine whether the integration of the separate elements happens in a theory consistent or inconsistent way. To quantify this process, the Integration Index (INTI) can be computed by simply adding all of the different theory-congruent associations between the separate elements within a certain range of sessions (e.g., sessions 5–10, sessions 60–65, etc.). As the depressive gains insight into the structure of his/her depressive experiences (i.e., as the above-described structure appears in the narratives of the depressive), we suppose that new – non-depressive – identifications will appear in the depressive’s narratives. To quantify this process, the Non-Depressive Identifications Index (NDII) can be computed by counting the references to such identifications in the transcriptions of the sessions. While we hypothesize a zero-correlation between the INTI and depressive complaints (as measured by the self-report questionnaires and the stress hormones), we hypothesize a significantly negative correlation between the NDII and depressive complaints (i.e., as the former increases, the latter will decrease). In general, four possible observations will lead to complete or partial rejection of the hypothesized structure: (1) The separate characteristics are not observed in the narrative material; (2) Theory-incongruent associations are observed between the separate characteristics; and (3) The INTI shows a negative correlation with measures of depressive complaints; and (4) The NDII does not show the predicted negative associations with measures of depressive complaints.

With respect to selecting participants for such studies, patients for whom depression is the primary complaint are the most suitable. Participants can be selected according to DSM-IV criteria, by means of the Structured Interview for DSM-IV diagnostics (SCID-II; First et al., 1997) and the Beck Depression Inventory (BDI-II; Beck et al., 1996). It is often the case that psychological problems are accompanied by a certain feeling of depression. In other words, depression can manifest as a side effect of another type of problem. While in such cases the mechanism of the depression might be similar to the one outlined above, these patients should not be considered suitable for studies of this kind. Studying subtypes of depression, such as the anaclitic and introjective types described by Blatt (1974), is essential in understanding the differential manifestation of depressive phenomenology in different personality structures, but it is of secondary importance to the investigation of the general mechanism of depression. Such subtypes are arguably variations of the general mechanisms of depression, effectuated by differences at the level of the broader personality organization.

Some authors argue that in *Mourning and Melancholia* Freud is referring to *psychotic* depression. This interpretation seems to be based on the fact that Freud used the term melancholia rather than depression. However, Freud’s remarks in the beginning of this text make clear that he was referring to depression in general, and not (only) psychotic depression. The use of the term melancholia was widespread in German psychiatry in the nineteenth century. Furthermore, in his well-known case-study of Dora, Freud makes clear that he considered melancholia treatable by means of psychoanalysis (Freud, 1905, p. 54). Bearing in mind that he did not consider psychoanalytic treatment appropriate for psychotic disorders, it seems highly improbable that he used the term melancholia with reference to psychotic depression.

Apart from diagnostic specifications, there are other restrictions in the selection of participants for single case-study research into depression. Not all depressive patients will be motivated to go through such an arduous and often long-term therapeutic process. Some patients will have a preference for another type of therapy, some might terminate the treatment before it reaches the point where the underlying structure is revealed. The application of our research procedure is therefore limited to the subgroup of patients that choose for psychoanalytic therapy. Whether this type of therapy is more effective in reducing depressive complaints than other types of therapy is a question that cannot be addressed in single case-study research (see also Desmet, 2013). What can be addressed, however, is the specific nature of the effects of psychoanalytic therapy and the process that leads to these effects. There are undoubtedly many roads toward recovery from depressive symptoms. The effects of the different types of therapy on the broader personality organization – or the subjectivity of the patient – however, might be quite different. Certain therapies, such as behavioral therapies, aim the straightforward restoration of the cohesion of the patient’s Ego, without making a detour to the unconscious or non-integrated aspects of the patient’s subjectivity; other therapies, such as psychoanalysis, do undertake this detour in order to place the patient in charge of his own life and to make him the author of his own history; still other therapies aim at a temporarily relief from depressing drives and ideations by means of pharmaceutical interventions to open up a pathway out of the depressive experiences. Whether or not interventions are successful probably depends on the match between the type of interventions and the characteristics of the patient’s personality.

## Discussion and Conclusion

In the first part of this study we presented a conceptual analysis of Freud’s theory of depression. In doing so, we highlighted the demonic side of depression, i.e., the sadistic drive. From a therapeutic perspective, it is important to situate the sadistic drive at the appropriate level. Depression – like any other type of sadism – is an ultimate attempt to exist for a significant other. When the subject does not exist for another through tenderness, he might do so by inflicting pain. Through the suffering of the other, the sadist finds confirmation that he/she exists for him/her. What is unbearable for the subject is the indifference of the other. In this respect, sadism is a derivate of love. Tracing back depressive sadism to its roots in love, reintegrating it in the subjective experience of love, might be the ultimate therapeutic effect.

This conceptual analysis resulted in a figure representing a schematic overview of Freud’s causal reasoning on depression. In the second part of this study, we presented a single case research design to put this theory to the test. In this design, we proposed to start from the identification of separate characteristics of depression in the clinical material. Subsequently, assessment of the global structure of depression can happen through an examination of the associations between the separate characteristics. From conceptual, clinical, and psychometric perspectives, I believe this research design is adequate for testing Freud’s theory on depression. It is without any doubt that the logical structure presented here will have to be adapted in confrontation with empirical material. Therefore, it cannot be considered an established body of knowledge, but only a fertile starting point for empirical research.

## Conflict of Interest Statement

The authors declare that the research was conducted in the absence of any commercial or financial relationships that could be construed as a potential conflict of interest.
